# Haploidentical Family Donor Transplantation: At the Crossroads of a Changing Paradigm

**DOI:** 10.1155/2016/9753542

**Published:** 2016-06-02

**Authors:** Suparno Chakrabarti, Franco Aversa, Yair Reisner, Paul O'Donnell

**Affiliations:** ^1^Department of BMT & Hematology, Dharamshila Hospital & Research Centre, New Delhi 110096, India; ^2^Manashi Chakrabarti Foundation, Kolkata 70032, India; ^3^Hematology and BMT Unit, University of Parma, 43126 Parma, Italy; ^4^Henry Drake Professorial Chair, Department of Immunology, Weizmann Institute of Science, 7610001 Rehovot, Israel; ^5^Massachusetts General Hospital Cancer Center, Boston, MA 02114, USA

The obvious advantage of haploidentical hematopoietic stem cell transplantation (HSCT) is that almost every patient who does not have HLA-identical donor or who urgently needs transplantation has at least one family member with whom to share one haplotype and who is promptly available as a donor. Over the past decade, haploidentical HSCT has spread to centers worldwide and several hundred patients have been treated even though significant variations on two main approaches have emerged over the past decade. Some centers have preferred an approach based on T cell depletion of G-CSF-mobilised peripheral blood progenitor cells (PBPCs); others have focused on manipulating the conditioning regimens and posttransplant immune suppression after the infusion of unmanipulated grafts.

The graft can be a megadose of positively/negatively T cell depleted or unmanipulated either bone marrow hematopoietic stem cells or PBPCs. Although haploidentical transplant modalities are based mainly on high intensity conditioning regimens, recently introduced reduced intensity regimens (RIC) showed promise in decreasing early transplant-related mortality (TRM) and extending the opportunity of hematopoietic stem cell transplantation (HSCT) to an elderly population with more comorbidities. Overall survival and clinical outcome continue to improve. Future challenges lie in determining the safest preparative conditioning regimen, minimizing GvHD while preserving effective GvL effect and promoting rapid immune reconstitution. Today, a haploidentical HSCT should be offered, not as a last resort, but as a viable option to patients who do not have, or cannot find, a matched donor.

In this special issue, ten articles have been selected to cover this emerging landscape. K. Parmesar and K. Raj extensively review the present status of haploidentical HSCT in adults with hematological malignancies, covering both T cell manipulated and unmanipulated approaches. This is further dwelt upon by two separate reviews by M. F. Zahid and D. A. Rizzieri and W. A. Fabricius and M. Ramanathan.

Posttransplantation cyclophosphamide (PTCy) has been widely used in the past few years for haploidentical HSCT in adults. A. Bacigalupo and S. Sica describe their experience with the use of PTCy and so do S. R. McCurdy and E. J. Fuchs. Both groups highlight the fact that the outcome of PTCy-based haploidentical HSCT is comparable to that of matched related or unrelated donors. Whilst the PTCy-based approach was based on RIC, S. R. Solomon et al. provide their perspective on myeloablative conditioning and PTCy with improved relapse-free survival.

Whilst PTCy was initially employed in conjunction with marrow graft, several groups have reported the same with mobilised PBPC grafts. S. Farhan et al. review the existing literature on the impact of graft source on the outcome of PTCy-based haploidentical HSCT. One of the major issues in haploidentical donor selection is the presence of anti-donor HLA antibodies which are associated with graft failure. P. Kongtim et al. discuss the importance of humoral immunity in graft rejection and possible therapeutic measures to address this problem.

The choice of alternative donor for HSCT in children with acute leukemia remains controversial. S. R. Jaiswal and S. Chakrabarti provide a perspective on the current place of haploidentical HSCT in children lacking a matched donor. They highlight the fact that extrapolation of outcome data from adults to children is not often linear and T cell manipulation might still be the preferred option in younger children undergoing haploidentical HSCT.

Finally, Y.-B. Chen et al. address the issue of transplantation tolerance and the possibility of HSCT and solid organ transplantation. In this context, they describe their experience with a protocol designed to enable induction of tolerance in SOT using a PTCy-based HSCT.

